# A cooperativity between virus and bacteria during respiratory infections

**DOI:** 10.3389/fmicb.2023.1279159

**Published:** 2023-11-30

**Authors:** C. Lalbiaktluangi, Mukesh Kumar Yadav, Prashant Kumar Singh, Amit Singh, Mahalaxmi Iyer, Balachandar Vellingiri, Ruth Zomuansangi, Zothanpuia, Heera Ram

**Affiliations:** ^1^Department of Microbiology, Central University of Punjab, Bathinda, Punjab, India; ^2^Department of Biotechnology, Mizoram University (A Central University), Pachhunga University College, Aizawl, Mizoram, India; ^3^Department of Zoology, Central University of Punjab, Bathinda, Punjab, India; ^4^Department of Zoology, Jai Narain Vyas University, Jodhpur, India

**Keywords:** viral infection, respiratory diseases, COVID-19, bacterial contamination, cooperative relation

## Abstract

Respiratory tract infections remain the leading cause of morbidity and mortality worldwide. The burden is further increased by polymicrobial infection or viral and bacterial co-infection, often exacerbating the existing condition. Way back in 1918, high morbidity due to secondary pneumonia caused by bacterial infection was known, and a similar phenomenon was observed during the recent COVID-19 pandemic in which secondary bacterial infection worsens the Severe Acute Respiratory Syndrome Coronavirus 2 (SARS-CoV-2) condition. It has been observed that viruses paved the way for subsequent bacterial infection; similarly, bacteria have also been found to aid in viral infection. Viruses elevate bacterial infection by impairing the host’s immune response, disrupting epithelial barrier integrity, expression of surface receptors and adhesion proteins, direct binding of virus to bacteria, altering nutritional immunity, and effecting the bacterial biofilm. Similarly, the bacteria enhance viral infection by altering the host’s immune response, up-regulation of adhesion proteins, and activation of viral proteins. During co-infection, respiratory bacterial and viral pathogens were found to adapt and co-exist in the airways of their survival and to benefit from each other, i.e., there is a cooperative existence between the two. This review comprehensively reviews the mechanisms involved in the synergistic/cooperativity relationship between viruses and bacteria and their interaction in clinically relevant respiratory infections.

## 1 Introduction

Respiratory tract infections are among the most prevalent human infections and are a major health and economic burden worldwide. They include upper respiratory tract infections (URTI) like pharyngitis, nasopharyngitis, otitis media, and tonsillitis and lower respiratory tract infections (LRTI) like pneumonia, tuberculosis, bronchitis, and bronchiolitis (Dasaraju and Liu, 1996). World Health Organization (WHO) estimated that 4 million people die each year due to acute respiratory infections (ARI), out of which the majority are infants, children, and geriatric patients (World Health Organization, 2020). In developed countries, it was estimated that respiratory infections account for 60% of antibiotic prescriptions (Lindbaek, 2006) and more than 30% of sick leave. The burden is much higher in developing countries where respiratory infections accounted for the majority of deaths in children below the age of 5 years (Bryce et al., 2005). Both bacteria and viruses have been found to cause respiratory infections in humans. *Streptococcus pneumoniae, Staphylococcus aureus, Haemophilus influenzae, Moraxella catarrhalis, Klebsiella pneumoniae*, and *Pseudomonas aeruginosa* are the most common bacterial pathogens detected in respiratory tract infections (Cappelletty, 1998; Burillo and Bouza, 2010; Gadsby et al., 2015). Viral pathogens like Influenza A (IAV) and B, Respiratory Syncytial Virus (RSV), Adenovirus, Parainfluenza viruses, and Human Bocavirus account for 30–40% of respiratory infections (Huang et al., 2013; Wen et al., 2019).

Recent technological advances have detected the presence of both viruses and bacteria in different respiratory diseases such as pneumonia, otitis media (OM), cystic fibrosis (CF), Coronavirus – 19 (COVID-19), etc. Research has suggested that high morbidity during the 1918 pandemic may be due to secondary pneumonia caused by bacterial infection (Morens et al., 2008). A similar phenomenon was observed during the recent COVID-19 pandemic in which secondary bacterial infection worsens the SARS-CoV-2 condition (Lansbury et al., 2020). De Bruyn et al. (2022) found that 68% of the COVID-19 patients admitted to the ICU developed secondary bacterial infections like pneumonia, bacteremia, etc. In a study conducted in Saudi Arabia, higher mortality rates were observed in COVID-19 patients co-infected with bacteria (50%) as compared to the SARS-CoV-2- only infected group (18.7%) (Alqahtani et al., 2022). Different studies have shown that there is a symbiotic interaction between viruses and bacteria to establish an infection. Bacteria have been found to aid viral infection by altering the host’s immune response (Bellinghausen et al., 2016), upregulating the surface adhesion proteins (Gulraiz et al., 2015), by activation of viral proteins (Tashiro et al., 1987a,b), etc. Similarly, viruses have been found to aid bacteria in establishing secondary infection through several mechanisms, including impairment of the host’s immune response (Ghoneim et al., 2013), disruption of epithelial barrier integrity (Short et al., 2016), expression of surface receptors and adhesion proteins (Avadhanula et al., 2006), direct binding of virus to bacteria (Rowe et al., 2019), alteration of nutritional immunity (Siegel et al., 2014), etc. For better management of infectious diseases, it is essential to understand the underlying mechanism by which bacteria and viruses interact and aggregate, infect, or establish infection. This review provides a comprehensive look into the mechanisms involved in viral and bacterial co-infections and how this interaction played out in different respiratory infections in human.

## 2 Search strategy

The review was conducted through searches of the PubMed database using different search terms. The search term “viral bacterial co-infection” yielded 3,289 results; the results were filtered to focus on co-infection in the respiratory tract and to eliminate co-infections in other systems like urinary tract infections and intestinal and gut infections. For the keyword “viral, bacterial co-infection,” the number of published articles increased significantly from 84 papers in 2010 to 315 papers in 2022. Other specific keywords were employed to study the co-infection patterns in different diseases. In total, 147 papers were reviewed in this study.

## 3 Interactions of bacteria and viruses in human infections

Viruses and bacteria can interact directly or indirectly in human infection, and interaction between these two could be either positive or negative (Almand et al., 2017). Mutualism, symbiosis, or aiding in the evasion of the host’s immune system are all examples of positive associations. At the same time, negative interactions occur through ammensalism, predation, or when the host immune system affects one organism over the other. The processes driving the synergy between virus and bacteria are complicated and multifaceted, with bacterial, viral, and host immunological components contributing to increased predisposition of infection. Many bacteria and viruses interact with each other and establish successful respiratory infections (Table 1). The respiratory viruses and bacterial interactions predispose bacterial superinfections, resulting in severe illness, and these interactions can influence microbial pathogenesis, including increased bacterial adhesion, enhanced virion stability, and modulation of the immune response by one microbe that benefits the other (Håkansson et al., 1994; El Ahmer et al., 1999). Similarly, bacterial infection has also been found to modulate viral infections exacerbating the disease (Verkaik et al., 2011; Smith et al., 2013; Nguyen et al., 2015).

**TABLE 1:** Mechanisms involved in viral predisposition to bacterial infection.

Mechanism	Example	Virus	Bacteria	References
Impairment of host’s immune response	Dysfunction and depletion of alveolar macrophage (AM)	Influenza virus	*Streptococcus pneumoniae*	Short et al., 2012a,b; Ghoneim et al., 2013
	Dysfunction and apoptosis of neutrophil	Respiratory syncytial virus	*Staphylococcus aureus*, *Pseudomonas aeruginosa*	Stark et al., 2006
	Reduced TNF- α induced NK cell production and impairing the activation of NK cells	H1N1 influenza virus	*Staphylococcus aureus*	Small et al., 2010
	Reduction of immune memory by reduced activation of naïve antigen-specific T-cells	RSV	-	González et al., 2008
	Decreased dendritic cell population in the lungs due to viral-induced decreased production of FLT3-L	Influenza A virus	*Streptococcus pneumoniae*	Beshara et al., 2018
	Inhibition of chemokine CCL2 production through enhanced type I IFN expression	Influenza virus	*Streptococcus pneumoniae*	Nakamura et al., 2011
	Decreased production of IL-17 and IL-22 cytokines	Influenza Virus	*Staphylococcus aureus*	Kudva et al., 2011
	Attenuation of Type 17 immunity by decreasing IL-1β production	Influenza Virus	*Staphylococcus aureus*	Robinson et al., 2013
Disruption of epithelial barrier integrity	Increased barrier permeability due to breakage of tight junctions (TJs)	IAV (H1N1 and H5N1 subtypes)	–	Short et al., 2016
	Impaired barrier integrity due to loss of ZO-1 protein from Tight Junctions	Rhinovirus	Non-typeable *Haemophilus influenzae* (NTHi)	Sajjan et al., 2006
	Reduction of transbarrier electrical resistance via the decreased expression of occludin and reduced function of Na + /K + ATPase pump	Human Bacovirus	–	Chiu et al., 2014; Balasubramaniam et al., 2015
Expression surface receptors and adhesion proteins	Exposure of carbohydrate ligands by cleavage of terminal sialic acid residues using neuraminidase	Influenza A and B	*Streptococcus pneumonia*	McCullers and Bartmess, 2003a
	Viral induced damage of epithelial cells increased expression of surface receptors PAFr ICAM-1, CEACAM-1, and P5 fimbriae	Influenza, parainfluenza and respiratory syncytial virus	*Streptococcus pneumoniae, Staphylococcus aureus, M.catarhallis*	Jiang et al., 1999; McCullers and Rehg, 2002; Ishizuka et al., 2003; Avadhanula et al., 2006
	Viral induced damage of epithelial cells increased expression of adhesion proteins; fibronectin and α5 integrin	Influenza, Parainfluenza and RSV	*Streptococcus pneumoniae, Staphylococcus aureus, Moxarella catarhallis*	Sajjan et al., 2006; Wang et al., 2009; Li et al., 2015
Direct binding of Virus to Bacteria	Binding of bacteria to viral G-protein	Respiratory Syncytial Virus	*Streptococccus pneumonia*	Hament et al., 2005
	Binding of RSV G-protein to penicillin binding protein 1a in bacteria	Respiratory syncytial virus	*Streptococccus pneumonia*	Smith et al., 2014
	Binding of viruses to bacterial capsular protein	Influenza virus, parainfluenza	Group B *streptococci* (GBS)	Tong et al., 2018a,b
		Influenza virus	Group A *Streptococci* (GAS)	Okamoto et al., 2004
Alteration of Nutritional Immunity	Increased availability of Iron through the reduction of production of an iron sequester Lipocalin -2	Influenza virus	*Staphylococcus aureus*	Robinson et al., 2013
	Increased availability of sialic acid and sialylated mucin through the breakdown of sialic acid residues	Influenza virus	*Streptococcus pneumonia*	Siegel et al., 2014
Effect on bacterial biofilm	Promotion of biofilm growth by increasing available iron	Respiratory syncytial virus	*Pseudomonas aeruginosa*,	Hendricks et al., 2016
	Promotion of biofilm growth by increasing available nutrients	Respiratory syncytial virus	*Staphylococcus aureus*	Kiedrowski and Bomberger, 2018
	Viral release of H_2_O_2_ facilitates the dispersion of biofilms	Rhinovirus (RV)	*Pseudomonas aeruginosa*	Chattoraj et al., 2011a
	Viral induced alteration of host environment promotes biofilm dispersal and translocation from nasopharynx to the lungs	Influenza A	*Staphylococcus aureus*	Reddinger et al., 2016
	Viral induced alteration of host environment promotes biofilm dispersal and translocation from nasopharynx to the lungs	Influenza A	*Streptococcus pneumonia*	Marks et al., 2013

### 3.1 Virus elevates bacteria invasion

**FIGURE 1:**
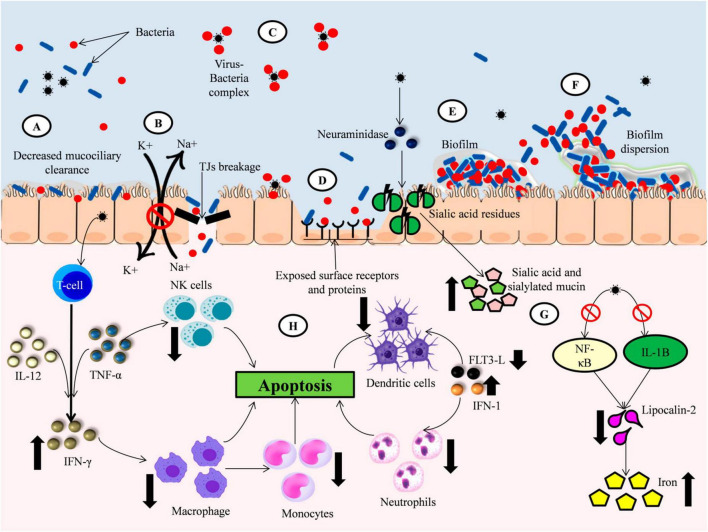
Elevation of bacterial colonization by viral infection. **(A)** Virus infection decrease the mucociliary clearance action resulting in increased bacterial colonization. **(B)** Viral infection impaired the epithelial barrier integrity by breaking Tight Junctions (TJs) and inhibiting Na+/K + ATPase pump promoting bacterial entry to the host cells. **(C)** Viruses directly binds to bacterial surface proteins forming a viral-bacterial complex that can infect uninfected cells as well as infected cells. **(D)** Viral proteins like Neuraminidase (NA) cleaves the sialic acid residues on the epithelial cell membrane exposing surface receptors and adhesion proteins aiding bacterial colonization. **(E)** Viral infection promotes bacterial biofilm formation and growth by providing increased nutrients. **(F)** Viruses induced changes in host environment promotes dispersion of bacterial biofilm leading to increased translocation of more virulent bacteria to other sites. **(G)** Viral infection induce change in nutritional immunity of the host resulting in elevated levels of nutrients which are utilized by bacteria for their growth. **(H)** Viral infection altered the host’s immune response to bacteria by decreasing the production and function of important immune cells like macrophages, NK cells, Dendritic cells and Neutrophils.

It has been established that prior viral infection augmented bacterial adherence to host cells. For example, it has been observed that in children with acute otitis media, prior infection by adenovirus type 1, 2, 3, and 5 significantly enhances pneumococcal colonization (Håkansson et al., 1994). Another study by El Ahmer et al. (1999) also found that influenza A infection of Hep-2 cells increased the adherence of *S. pneumonia, H. influenza*, and *M. catarrhalis* to virus-infected Hep-2 cells. Virus-induced bacterial adherence is achieved through several mechanisms, which are briefly discussed in this section. The mechanisms are represented and summarized in Figure 1 and Table 1, respectively.

(i) Impairment of the host’s immune response:

Respiratory viruses can disrupt the host’s immune system through the malfunction and depletion of innate immune cells. One of the most well-studied cases is the influenza virus-induced dysfunction and depletion of alveolar macrophages, promoting subsequent bacterial infection (Ghoneim et al., 2013; Short et al., 2016). RSV infection has been found to cause neutrophil dysfunction by lowering oxidative burst and promoting apoptosis of neutrophils, elevating the adherence of *S. aureus* or *P. aeruginosa* (Stark et al., 2006). Influenza infection also modifies monocyte functions, inhibiting proper immune response and reducing cytokine activity and synthesis, resulting in increased bacterial colonization and mortality risk (Abramson et al., 1982). Another innate immune cell affected by viral infection is Natural Killer (NK) cells, which are the critical components of the innate immune response. Small et al. (2010) found that influenza infection increased susceptibility to *S. aureus* by reducing TNF-α induced NK cell production and impairing the activation of NK cells. Viral infection also affects the adaptive immune response like T CD8 + and T CD4 + cell and antibody responses. RSV has been found to facilitate reinfection by reducing immune memory. This is achieved by impairing the interaction between dendritic cells and T-cells, reducing activation of naïve antigen-specific T-cells (González et al., 2008). In influenza-infected mice, a decrease in the production of FMS-like tyrosine kinase 3 ligand (FLT3-L), a dendritic cell differentiation factor was observed, resulting in decreased dendritic cell population in the lungs contributing to secondary bacterial infection (Beshara et al., 2018).

Depletion and dysfunction of innate and adaptive immune cells significantly affect the production of cytokines and chemokines. Influenza infection has been found to decrease the production of pro-inflammatory cytokines like IL-1β, IL-6, and TNF-α, decreasing bacterial clearance. Influenza infection has also been found to affect the adaptive Type 17 response. Nakamura et al. (2011) found that influenza inhibited the production of chemokine CCL2 responsible for bacterial clearance through the enhanced expression of type I IFN, thus promoting pneumococcal colonization. Robinson et al. (2013) also found that Influenza infection exacerbates *S. aureus* infection by decreasing IL-1β production, which attenuates Type 17 immunity. Lastly, Kudva et al. (2011) found that in mice infected with influenza virus and *Staphylococcus aureus*, a significant decrease in production of IL-17 and IL-22 cytokines was observed. Interestingly, cytokine release during the acute inflammation stage has been found to be capable of enhancing the growth of bacteria and prolonging bacterial infection (dos Santos et al., 2002). Kanangat et al. (1999) and Meduri et al. (1999) found that high concentrations of cytokines like IL-1b, IL-6, and TNF-α enhance the *in vitro* growth of bacteria, including *S. aureus*, *P. aeruginosa*, and *Actinobacteria* sp.

(ii) Disruption of epithelial barrier integrity

Viral infection has been found to cause extensive damage to the human epithelial and endothelial cells. Epithelial barrier damage may occur due to direct cleavage of tight junctions. Downregulation of junction proteins through the host’s pro-inflammatory response against the virus results in impaired barrier integrity, increased membrane permeability, and decreased trans-barrier electrical resistance (Rossi et al., 2020). Tight Junctions (TJ) act as a sealant between neighboring cells and maintain the integrity of the airway epithelial cells (Steed et al., 2010). Viral infections induce the breakage of tight junctions (TJs), which increases the permeability of molecules in the airway epithelial cells, facilitating the entry and transmigration of bacteria in the airway (Short et al., 2016). ZO-1 protein in TJ established a link between the transmembrane protein occludin and the actin cytoskeleton and plays an essential role in maintaining the structural integrity of TJs (Itoh et al., 1997). When infected by Rhinovirus, loss of ZO-1 protein was observed, which facilitates the entry of Non-typeable *Haemophilus influenzae* (NTHi) (Sajjan et al., 2008). Infection by human bocavirus has also been found to reduce transbarrier electrical resistance via the decreased expression of occludin and reduced function of the Na + /K + ATPase pump (Chiu et al., 2014; Balasubramaniam et al., 2015). Viral infection may also cause denudation of the epithelial layer by killing the epithelial cells through direct lysis or metabolic exhaustion (Bosch et al., 2013).

(iii) Expression of surface receptors and adhesion proteins

Viruses have been found to facilitate bacterial adherence by exposing cell surface receptors on the epithelial cells. Influenza A and B exposed the carbohydrate ligands on the epithelial cells by cleaving the terminal sialic acid residues using neuraminidase. The exposed carbohydrate ligands then act as a binding site for *S. pneumoniae* (McCullers and Bartmess, 2003a; Li et al., 2015). Inflammatory response to influenza, parainfluenza, and RSV infection caused epithelial injury resulting in increased expression of cell surface receptors such as platelet-activating factor receptor (PAFr), intercellular adhesion molecule-1 (ICAM-1), which serves as bacterial receptors (Ishizuka et al., 2003; McCullers and Bartmess, 2003b; Avadhanula et al., 2006). Upregulation of other surface receptors, such as carcinoembryonic adhesion molecule-1 (CEACAM-1), and P5-homologous fimbriae (P5 fimbriae) were also observed (Jiang et al., 1999). Studies showed that pathogenic bacteria like *S. pneumoniae, S. aureus*, and *M.catarhallis* could strongly bind to fibronectin and α5 integrin, which were exposed after viral-induced cell injury (Sajjan et al., 2006; Wang et al., 2009). Viruses also induce bacterial adherence through the increased expression of viral surface proteins, which are known receptors for bacteria.

(iv) Direct binding of the virus to bacteria

The role played by viral glycoproteins and surface receptors on enhancing bacterial adherence is well established. This can explain the increased adherence to virus-infected cells but doesn’t fully explain the increased bacterial adherence to uninfected cells. An alternative mechanism has been introduced where the virus directly binds to the incoming bacteria, thus explaining the bacterial adherence not only to infected cells but also to uninfected cells. The hypothesis is that virus binding to bacteria acts as a coupling agent between the uninfected epithelial cells and bacteria, leading to increased bacterial adherence. Hament et al. (2005) found that G-protein produced by RSV facilitates the binding to *S. pneumoniae*, after which the pneumococcal adherence to uninfected epithelial cells is significantly increased. Smith et al. (2014) found that the G-protein of RSV binds to penicillin-binding protein 1a of *S. pneumoniae*, increasing the virulence of *S. pneumoniae*. Viruses like influenza and parainfluenza viruses recognize the capsular polysaccharides expressed by Group B *streptococci* (GBS) and bind to the bacteria, enhancing bacterial adherence (Tong et al., 2018a,b). Okamoto et al. (2004) found that the influenza virus can bind to encapsulated Group A *Streptococci* (GAS), facilitating bacterial adherence to virus-infected alveolar epithelial cells. Similarly, Rowe et al. (2019) studied the direct interaction between the influenza virus and Gram-positive bacteria like *S. pneumoniae* and *S. aureus*, as well as Gram-negative bacteria, *M. catarrhalis*, and non-typeable *Haemophilus.* They incubated the influenza virus with bacteria, and increased bacterial adherence was observed even after the removal of unbound viruses. The same phenomenon was also observed in *in vivo*, where higher bacterial adherence was observed in murine tissues infected with bacteria pre-incubated with viruses compared to normal bacteria (Rowe et al., 2019).

(v) Increased availability of nutrients

In healthy cells, the minerals, growth factors, and other nutrients needed by the bacteria are sequestered by the host to prevent pathogenicity in a phenomenon called nutritional immunity (Hennigar and McClung, 2016). This phenomenon is altered when viruses infect the host cells, promoting further infection by bacteria. Influenza virus infection has been found to reduce the production of an iron sequester, Lipocalin -2, by suppressing the activation of NF-kB and expression of IL-1B. This increased iron availability in the host, exacerbating *S. aureus* acute pneumonia in mice (Robinson et al., 2013). Influenza virus infection also increases the availability of sialic acid and sialylated mucin in the host, stimulating pneumococcal growth in the airway (Siegel et al., 2014). RSV infection has also been found to increase iron concentration in the host, promoting *P. aeruginosa* biofilm formation (Hendricks et al., 2016). In another study, Hendricks et al. (2016) also found that the extracellular vesicles released from the virally infected cells act as a transferrin transport vehicle between cells, enhancing the growth of *P. aeruginosa* biofilm. The concentration of other nutrient sources like mucins and surfactant proteins in the host was also altered during virus co-infection (Kiedrowski and Bomberger, 2018).

(vi) Effect on bacterial biofilm

It has been observed that viruses induce the transition of bacterial growth to a biofilm mode by altering nutrient immunity (Hendricks et al., 2016; Kiedrowski et al., 2018). Using a co-culture model, Hendricks et al. (2016) found that RSV infection-induced IFN signaling stimulates the release of iron-bound transferrin protein, which promotes thriving biofilm formation by *P. aeruginosa*. Similarly, Kiedrowski et al. (2018) found that RSV infection enhanced *S. aureus* biofilm formation due to the increased release of nutrients by the host. They also found that *S. aureus* genes involved in protein translation and transport, growth, and amino acid metabolism were upregulated during co-infection.

Biofilm is the reservoir of the bacteria, and the dispersion plays a vital role in the pathogenesis and progression of a disease. Viral infections have been found to influence bacterial biofilm dispersion and its transition from asymptomatic colonization to invasive disease. In Cystic Fibrosis patients, Rhinovirus induces the release of H_2_O_2_, which facilitates the dispersion of *P. aeruginosa* biofilms (Chattoraj et al., 2011a). Reddinger et al. (2016) found that *S. aureus* biofilms on the upper respiratory tract dispersed due to changes in the host induced by influenza infection. The biofilm dispersion induces the translocation of cells from the nasal tissue to the lungs in a mice model, elevating the progression of pneumonia. Marks et al. (2013) observed the same phenomenon in which influenza A infection induced the dispersion of *S. pneumoniae* biofilm. The dispersed cells were found to have different phenotypes with upregulation of different virulence genes, exacerbating disease progression (Marks et al., 2013; Pettigrew et al., 2014).

### 3.2 Bacteria elevates viral invasion

**FIGURE 2:**
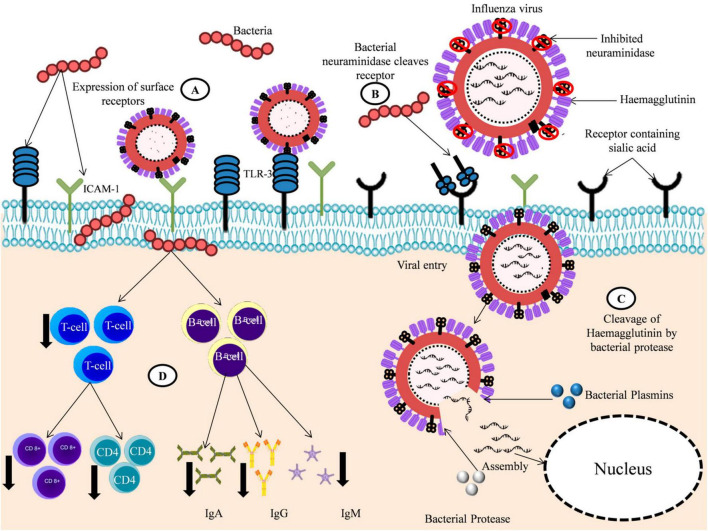
Bacteria aids in viral infection. **(A)** Bacterial infection increased the expression of surface proteins like ICAM-1 and TLR-3 aiding the attachment of viruses to host cells. **(B)** Bacterial Neuraminidase (NA) aids in the viral entry to host cells by cleaving sialic acid receptors when viral Neuraminidase (NA) are inhibited. **(C)** Bacterial proteases and plasmins aids in the release of viral RNA by cleaving the viral protein Haemaglutinin (HA). **(D)** Bacteria alters the immune response to virus by decreasing the production of viral-specific immune cells CD8+, CD4+, and antibodies like IgG, IgA, and IgM.

Multiple studies have been carried out to study the role of bacteria on viral pathogenesis with varying results. Some studies found that bacterial infection negatively impacts viral survival and growth (Wolf et al., 2014); however, most studies showed a positive correlation between bacteria and viruses (Blevins et al., 2014; Wu et al., 2015). Verkaik et al. (2011) found that prior infection of human bronchial epithelial cells by *S. pneumoniae* renders the cells more susceptible to hMPV infections. Nguyen et al. (2015) carried out *in vitro* and *in vivo* studies to observe the effect of *S. pneumoniae* on RSV infection and found that *S. pneumoniae* enhances RSV infection in both *in vitro* and *in vivo* studies. Similarly, Smith et al. (2013) also observed increased Influenza load in the presence of *S. pneumoniae*, which suggests that bacteria aids in the release of Influenza virus from airway cells as well as the release of virions. Bacteria-mediated modulation of viral infections is achieved mainly through the bacterial influence on the host and the host’s immune system, as well as its direct effect on the virus itself (Figure 2 and Table 2), which are briefly discussed below:

**TABLE 2:** Mechanisms involved in bacterial modulation of viral infection.

Mechanism	Example	Bacteria	Virus	References
Alteration host’s immune response	Decrease in the production and distribution of CD8 + T cell in the lung enhancing viral survival	*Streptococcus pneumoniae*	Influenza A virus	Blevins et al., 2014
	Decreased levels of viral-specific antibodies IgA, IgM and IgG increasing viral survival	*Streptococcus pneumoniae*	Influenza virus	Wu et al., 2015
	Decreased population of immune cells such as CD4 T cells, B cells, and plasma cells promoting viral survival	*Streptococcus pneumoniae*	Influenza A virus	Wu et al., 2015
Upregulation of adhesion proteins	Increased expression of intracellular adhesion molecules; ICAM-1 and TLR3	*Hemophilus influenzae*	Rhinovirus (RV)	Sajjan et al., 2006
	Upregulation of adhesion molecule ICAM-1	non-typable *Hemophilus influenzae* (nTHi)	Rhinovirus (HRV16)	Gulraiz et al., 2015
Activation of viral proteins	Production of proteases capable of cleaving and activating viral haemagglutinin (HA)	*Staphylococcus aureus, Aerococcus viridans*	Influenza A virus	Tashiro et al., 1987a,b; Scheiblauer et al., 1992
	Production of plasminogens (staphylokinase and streptokinase) capable of cleaving and activating viral haemagglutinin (HA)	*Staphylococcus aureus, Streptococcus pneumoniae*	H1N1 influenza virus	Tse et al., 2013
	Synthetic bacterial lipopeptide Pam3CSK4 aids in viral infection	–	RSV, measles virus, HIV-1, and human metapneumovirus	Nguyen et al., 2010
	Production of bacterial Neuraminidase (NA) capable of releasing virions in the absence of viral Neuraminidase (NA)	*Streptococcus pneumoniae*	Influenza virus	Nishikawa et al., 2012

(i) Alteration of the host’s immune response

Bacterial co-infection has been found to significantly influence a host’s immune response against viral infection. In most cases, these bacteria-modulated immune changes support viral infection, but some studies found adverse effects on viruses. Wolf et al. (2014) found that co-infection with *S. pneumoniae* elevates the B-cell response to the Influenza virus. Bellinghausen et al. (2016) also observed that although bacteria altered the airway immune response, it does not necessarily affect the subsequent viral infection. However, the contrasting results were reported by Blevins et al. (2014), which showed that prior infection by *S. pneumoniae* markedly decreased the lung’s anti-viral CD8 + T cell population, resulting in increased survival of the Influenza A virus and increased mortality. Using a murine mouse model, Wu et al. (2015) also found that secondary infection by *S. pneumoniae* significantly increased viral titers and decreased the concentration of IgA, IgM, and IgG, which are viral specific antibodies. They also detected a decrease in the population of immune cells, such as CD4 T cells, B cells, and plasma cells, facilitating viral survival in the host.

(ii) Up-regulation of adhesion proteins

Bacteria are also involved in up-regulation of the surface protein through altered immune response. Sajjan et al. (2006) found that *H. influenzae* infection increased the expression of intracellular adhesion molecules like ICAM-1 and TLR3, leading to increased infection by RV and subsequent RV-induced increased cytokine release (Sajjan et al., 2006). Similar results were obtained by Gulraiz et al. (2015), who found that prior non-typable *H. influenzae* infection dramatically increases the binding of HRV16 to two bronchial epithelial cell lines pBECs and BEAS-2B, most probably through the upregulation of ICAM-1. However, no such increase in binding was detected in HRV1B and RSV infection.

(iii) Activation of viral proteins

Various components of bacteria have been found to aid viral attachment and infection. Proteases produced by *S. aureus* have been found to increase the replication of various influenza strains in the lungs by cleaving and activating the viral haemagglutinin (HA) (Tashiro et al., 1987a,b). The activation of hemagglutinin was found to be strain-dependent, i.e., proteases produced by one bacterium work only for specific viral strains. Similarly, Scheiblauer et al. (1992) also found that protease secreted by a pathogen *Aerococcus viridans* could cleave the HA of various influenza A virus isolates, causing severe pneumonia in mice and leading to mortality. Other bacterial proteases that cannot directly cleave viral haemagglutinin (HA) have also been found to increase the viral load. Simultaneous administration of Influenza A virus and non-cleaving proteases secreted by *P. aeruginosa* has markedly increased the viral load (Scheiblauer et al., 1992). Although the specific proteases that can cleave viral HA from bacteria are yet to be identified, these proteases might provide a novel drug target for treating viral and bacterial co-infection. Plasminogen activators of *S. aureus* and *S. pneumoniae*, like staphylokinase and streptokinase, could generate plasmins, an important fibrinolytic protease. These plasminogens could cleave the hemagglutinin of H1N1 Influenza virus that has Ser-Tyr substitution in their cleavage site (Tse et al., 2013). The commensal bacteria of the upper and lower respiratory tract have been found to secrete neuraminidase, which can aid in viral infection. For example, Nishikawa et al. (2012) found that the neuraminidase secreted by *S. pneumoniae* can facilitate the successful release of virions even after the inhibition of viral neuraminidase. This can potentially hinder the efficacy of virus neuraminidase drugs like oseltamivir and zanamivir used during influenza infection.

Apart from the cleavage of viral proteins, bacterial peptides have been found to enhance viral infection by directly binding to the virus. A synthetic bacterial lipopeptide Pam3CSK4 has been found to enhance the infection of primary epithelial, lymphoid, and myeloid cells by RSV, measles virus, HIV-1, and human metapneumovirus independent of TLR activation. Pam3CSK4 contains two important cationic structures, N-palmitoylated cysteine and cationic SK4 sequence, which may interact directly with the negatively charged structures of the viral envelope or the host cells, enhancing the viral infection (Nguyen et al., 2010).

## 4 Major human infectious diseases and viral - bacteria co-infection

The use of molecular assays like PCR to assess clinical samples has provided a better picture of the synergy between bacteria and viruses in the pathogenesis of various infectious diseases (Ruuskanen et al., 2011). Co-infection has been observed in various infectious diseases with mostly adverse patient outcomes. Johansson et al. (2011) found that co-infection in CAP often increases the severity of CAP and results in more extended hospitalization. It has been suggested that a majority of deaths during the “Spanish Flu” pandemic may be due to the development of secondary pneumonia (Morens et al., 2008). Similarly, during the 2009 H1N1 pandemic, 29–55% of mortalities were attributed to secondary pneumonia (Center for Disease Control and Prevention [CDC], 2012). This is again observed during the most recent global COVID-19 pandemic, where secondary infection by bacteria worsens the severity of the disease (Langford et al., 2020). Interestingly, many epidemiological studies detected no pathogens in some patients (Huijskens et al., 2013; Luchsinger et al., 2013; Jain et al., 2015; Voiriot et al., 2016). For example, an epidemiological study on CAP adult patients requiring hospitalization in the US detected no pathogens in the majority of the patients. However, they detected co-infection in 38% of the patients (Jain et al., 2015). Another study by Luchsinger et al. (2013) also detected no pathogenic agents in 35% of the patients tested. The viral and bacterial pathogens co-infections detected in various respiratory diseases are summarized in Table 3.

**TABLE 3:** Some human diseases in which bacteria and viral co-infection were detected.

Disease	Virus	Bacteria	No. of patients	Percentage of co-infection	References
Pneumonia	Influenza virus	*Staphylococcus aureus, Streptococcus pneumonia*	9,860 children	3.1%	Williams et al., 2011
	Influenza A H1N1 virus	*Streptococcus pneumonia, Streptococcus pyogenes, Staphylococcus aureus, Mycoplasma pneumonia, Escherichia coli, Pseudomonas aeruginosa, Moraxella catarrhalis, Coxiella burnetii, Fusobacterium* sp.	128 adult patients	33%	Cillóniz et al., 2012
	Rhinovirus, Human Metapneumovirus, Human Bacovirus	*Streptococcus pneumoniae, Moraxella catarrhalis, Haemophilus influenzae*	76 children	66%	Honkinen et al., 2012
	Influenza A, Influenza B, respiratory syncytial virus, parainfluenza, coronavirus, adenovirus, bacovirus, human metapneumovirus	*Streptococcus pneumonia, Staphylococcus aureus, Haemophilus influenza, Chlamydia pneumonia, Enterobacteriaceae* sp.*, Pseudomonas aeruginosa, Legionella pneumophila*	174 adults	26%	Voiriot et al., 2016
Otitis Media	Parainfluenza virus 3 and enterovirus	*Staphylococcus aureus*	79 children	66%	Ruohola et al., 2006
	Enterovirus and rhinovirus	*Pseudomonas aeruginosa*			
	Enterovirus, coronavirus and rhinovirus	*Streptococcus pneumonia, Haemophilus influenza*			
	Respiratory syncytial virus, enterovirus, NT picornavirus	*Streptococcus pneumonia, Moraxella catarrhalis*			
	Enterovirus	*Haemophilus influenza, Moraxella catarrhalis*			
	Parainfluenza virus 3 and influenza A virus	*Streptococcus pneumonia, Haemophilus influenza, Moraxella catarrhalis*			
	Rhinovirus, Respiratory syncytial virus, NT Picornavirus, Human metapneumovirus	*Streptococcus pneumonia*			
	Rhinovirus, bacovirus, NT Picornavirus, Parainfluenza virus 3	*Haemophilus influenza*			
	Rhinovirus, Respiratory syncytial virus, Influenza A virus, Human Bacovirus, NT Picornavirus, coronavirus, Parainfluenza virus 3	*Moraxella catarrhalis*			
	Rhinovirus	*Streptococcus pneumonia, Streptococcus pyogenes*			
	Rhinovirus, Adenovirus, Bocavirus	*Streptococcus pneumoniae, Haemophilus influenzae, and Moraxella catarrhalis*	194 children	–	Pettigrew et al., 2011
Bronchiolitis	Respiratory Syncytial virus (RSV)	*Haemophilus influenzae, Staphylococcus aureus* *Moxarella catarrhalis Streptococcus pneumonia Streptococcus pyogenes, Pseudomonas aeruginosa*, *Bordetella pertussis*, *Klebsiella pneumonia*, *Escherichia coli*, *Enterobacter cloacae, Citrobacter freundii*, *Proteus mirabilis*, *Streptococcus agalactiae*, *Neisseria meningitides*, Methicillin Resistant *Staphylococcus aureus* (MRSA)	165 children	21.8%	Thorburn et al., 2006
	Respiratory Syncytial virus (RSV)	*Haemophilus influenzae, Staphylococcus aureus* *Moxarella catarrhalis Streptococcus pneumonia, Enterobacteriaceae*	167 children	37.7%	Wiegers et al., 2019
Cystic Fibrosis	Human Rhinovirus, Influenza A and B, Respiratory syncytial virus, Parainfluenza virus 1,2 and 3,Human Metapneumovirus	*Pseudomonas aeruginosa, Streptococcus pneumoniae Haemophilus influenza, Burkholderia cepacia*	37 patients	73%	Stelzer-Braid et al., 2012
	Rhinovirus, Adenovirus, Enterovirus, Parainfluenza viruses, Influenza viruses, Respiratory syncytial virus, Metapneumovirus B, Bocavirus	*Staphylococcus aureus, Haemophilus influenzae, Pseudomonas aeruginosa, Klebsiella oxytoca, Moraxella catarrhalis, Enterobacter cloacae, and Serratia marcescens.*	368 patients	14.7%	Miró-Cañís et al., 2017
COVID-19	SARS-CoV-2	*Chlamydophila pneumonia and*	117 patients	30%	Zahariadis et al., 2006
		*Mycoplasma pneumoniae*	117 patients	9%	
	SARS-CoV-2	*Staphylococcus aureus, Haemophilus influenza, Streptococcus pneumonia, Moraxella catarrhalis, Streptococcus agalactiae*	56 patients	10.6%	Kreitmann et al., 2020

Multiple tests have been used to detect the type of bacterial and viral species involved in various respiratory infectious diseases, including bacterial cultures, PCR, etc. After the first US FDA approval of respiratory syndromic panel in 2011, syndromic panels emerged as a quick and efficient way of detecting bacteria and viruses in co-infection patients. Currently, eight multiplex upper respiratory panels and two multiplex lower respiratory panels have been approved by the FDA for the diagnosis of upper and lower respiratory tract infections. An example of a URT disease syndromic panel is Applied BioCode Respiratory Pathogen Panel (RPP), which can detect three bacteria: *Bordetella pertussis*, *Chlamydia pneumoniae* and *Mycoplasma pneumoniae* and at least five viral species including Influenza A and B, Human metapneumovirus, RSV A and B, Parainfluenza virus 1, 2, 3, and 4, Human Rhinovirus in 4 h. Another syndromic panel, BioFire FilmArray Respiratory Panel EZ (CLIA-waived), can detect three bacterial species and eight viral species, including Coronavirus, in just 1 h. Multiplex lower respiratory panels BioFire FilmArray Pneumonia *plus* Panel and Curetis Unyvero Lower Respiratory Tract Panel can detect many bacterial and viral pathogens. The bacterial pathogens that these panels can detect include *S. aureus*, *Stenotrophomonas maltophilia*, *S. pneumoniae, P. aeruginosa.* These lower respiratory panels can detect viruses like Influenza A and B, Coronavirus, RSV, Adenovirus, and Parainfluenza virus. In addition to detecting the pathogens, multiplex lower respiratory panels can also be used for the detection of antibiotic resistance genes like Methicillin resistance genes: mecA/mecC and MREJ, Penicillin resistance gene; TEM (Bard and McElvania, 2020; Dumkow et al., 2021). False positive cultures can be a significant problem in clinical settings. Using strict, standardized criteria for sample collection, culture, and immunological procedures is required to determine whether the collected bacterial or viral pathogens are true pathogens, not contaminants or innocent bystanders.

### 4.1 Pneumonia

Pneumonia, a severe inflammation of the lungs, is one of the most commonly known respiratory tract infections responsible for the loss of thousands of lives each year (World Health Organization, 2013). It is the leading cause of mortality in children under 5 years old and attributed to total mortality of 2.5 million people in 2019 (Liu et al., 2012). Either viruses or bacteria cause pneumonia and sometimes fungi; however, bacteria is the primary causative agent in most cases. *S. pneumonia* and RSV are the most common bacterial and viral causes of pneumonia (Musher et al., 2017).

#### 4.1.1 Virus and bacteria cooperativity in pneumonia

Co-infection by viruses and bacteria is highly prevalent in community-acquired and hospital-acquired pneumonia (CAP). It has been reported that 45% of CAP cases in children are due to viral and bacterial co-infection, while reported cases of mixed infection in CAP cases in adults are lower (Ruuskanen et al., 2011). Cillóniz et al. (2012) studied the incidence of co-infection in CAP patients and detected bacterial co-infection in 33% of the patients.

Most reported cases of viral-bacterial pneumonia involved *S. pneumoniae* with different viruses. Other bacterial species like *S. aureus, P. aeruginosa, Neisseria meningitides, H. influenza*, and *K. pneumoniae* were also detected in secondary infection. A combination of the influenza virus and *S. pneumoniae* is the most frequent combination of co-infection (Handel et al., 2010; Honkinen et al., 2012). Before the prevalence of COVID-19, the most commonly found viral pathogens in community-acquired pneumonia patients were RSV, metapneumovirus, and influenza virus (Mandell, 2015). However, after and during the COVID-19 pandemic, pneumonia patients were detected with respiratory pathogenic bacteria and viruses, indicating that the interplay of respiratory bacteria and viruses is a major risk in pneumonia (Mirzaei et al., 2020; Ladas et al., 2023; Zhang et al., 2023).

The synergism between Influenza virus and *S. pneumoniae* in pneumonia involved several mechanisms, including altered immune response, epithelial barrier damage (Nita-Lazar et al., 2015), expression of surface receptors (McCullers and Rehg, 2002), impaired mucociliary clearance, dispersion of biofilm to sterile sites (Marks et al., 2013; Pettigrew et al., 2014), etc. which are explained in detail in Section 3. However, in addition to facilitating pneumococcal colonization/disease, recent studies have found that Influenza virus and other viruses play an important role in the transmission of pneumococci. In animal studies involving mice and ferrets, IAV infection has been found to facilitate intra- and inter-cage transmission of pneumococci (Diavatopoulos et al., 2010; McCullers et al., 2010). In mice, Diavatopoulos et al. (2010) found that pneumococci transmission occurred when all of the mice were infected with IAV, and transmission was prevented when IAV replication was inhibited in contact (uncolonized) or index (those colonized with *S. pneumoniae*) mice. In ferrets, McCullers et al. (2010) found that contact ferrets have a higher chance of acquiring pneumococcal infection when they are infected with IAV. Short et al. (2012a,b) investigated the factors determining the virus-mediated transmission of *S. pneumoniae* and found that elevation of the nasopharyngeal bacterial load in the index mouse and induction of inflammation in the contact mice facilitated pneumococcal colonization. Epidemiological studies on the role of viruses in the transmission of pneumococci found polarizing results. While some studies (Kuster et al., 2011) found no relationship between influenza seasonality and pneumococcal transmission, other studies (Grijalva et al., 2014; Karppinen et al., 2017) found that viruses like Influenza, Rhinovirus, and Parainfluenza infection facilitate the acquisition and transmission of *Streptococcus pneumoniae*.

### 4.2 Otitis media

Otitis media (OM) is a highly prevalent pediatric disease mainly affecting children under five. A prospective cohort study showed that 80% of children experience OM in childhood, one of the most common reasons for a doctor visit (Chonmaitree et al., 2008). It occurs due to impaired fluid drainage from the middle ear chamber, which facilitates the colonization of the middle ear by commensal bacteria residing in the nasopharynx (Vergison et al., 2010).

#### 4.2.1 Virus and bacteria cooperativity in otitis media

Concurrent infection by bacteria and viruses was observed in most OM cases (Vesa et al., 2001; Ruohola et al., 2006; Pettigrew et al., 2011). A cohort study on children aged 2–24 months detected with acute OM showed that most children had viral upper respiratory infections before being diagnosed with acute OM (Vesa et al., 2001). Ruohola et al. (2006) conducted a study to determine the microbiology of acute OM in children and detected viral bacterial co-infection in most (66%) patients. OM is a polymicrobial disease caused by a wide variety of viruses and bacteria, including *S. pneumonia, H. influenza, and M. catarrhalis*, and viruses like Adenovirus, Rhinovirus, and respiratory syncytial viruses (Chonmaitree et al., 2008). Among bacteria, non-typeable *H. influenza* was the most common among those pathogens (Vergison et al., 2010).

Several mechanisms involved in the cooperative relationship between bacterial pathogens of otitis media and viruses have been identified, including expression of adhesion molecules and receptors in the cells (Nita-Lazar et al., 2015), inflammatory injury, etc. (McNamee and Harmsen, 2006; Spelmink et al., 2016). A mechanism of viral, bacterial synergism unique to otitis media is the virus-induced dysfunction of the Eustachian tubes in the middle ear. Giebink et al. (1987) studied the effect of influenza virus infection on Eustachian tubes. They found that the viral infection induced inflammation of the tympanic membrane and the epithelium of the Eustachian tube. This resulted in an under-pressured middle ear that facilitates the entry of pathogens into the middle ear cavity (Bakaletz, 2017). Virus infections also affect the mucociliary clearance of middle ear fluid, making the middle ear much more susceptible to secondary bacterial infection (Park et al., 1993).

### 4.3 Bronchiolitis

Bronchiolitis is an infection of the lower respiratory tract commonly affecting children below 2 years. It is considered one of the most frequent causes of infant hospitalization and is often associated with high infant morbidity. Respiratory Syncytial Virus (RSV) was the primary cause of Bronchiolitis and was detected in more than 70% of the patients (Smyth and Openshaw, 2006). Other viruses, such as Parainfluenza virus (PIV), Influenza virus, Rhinovirus (RV), and Adenovirus, were also often detected in bronchiolitis patients (Nascimento et al., 2010). Although bronchiolitis is frequently associated with viruses, recent etiological studies also reported the presence of bacterial pathogens in patients (Thorburn et al., 2006; Wiegers et al., 2019). Wiegers et al. (2019) conducted a study on ventilated bronchiolitis patients and detected bacterial co-infection in more than one-third (37.7%) of the patients. They also found that patients with bacterial co-infection require more prolonged mechanical ventilation and PICU stay (Wiegers et al., 2019). Similarly, Thorburn et al. (2006) concluded that patients with bacterial co-infection require more extended hospital stays and detected bacterial co-infection in almost half (40%) of the patients. The most frequently isolated pathogens are *H. influenzae, S. pneumoniae, M.catarrhalis*, and *S. aureus*. However, other bacterial pathogens like *P. aeruginosa, K. pneumoniae, E. coli*, etc., were also isolated from some patients’ samples (Thorburn et al., 2006; Fares et al., 2011; Wiegers et al., 2019).

### 4.4 Cystic fibrosis

Co-infection, especially bacterial co-infection, is commonly found in Cystic Fibrosis (CF), a fatal genetic disease caused by Fibrosis Transmembrane Conductance Regulator (CFTR) gene mutations. CF is characterized by a progressive decline of pulmonary function due to the accumulation of thick mucus in the lungs and persistent chronic infection (Nichols et al., 2008; Scott et al., 2020). A wide variety of pathogenic bacteria, viruses, fungi, and sometimes yeast is involved in chronic infection of the lungs (Skov et al., 2005; Pihet et al., 2009; Sudfeld et al., 2010; Sherrard et al., 2014). Among these pathogens, *P.aeruginosa* is the most common cause of death in CF (Kiedrowski et al., 2018). The most frequently isolated pathogen from pediatric CF patients is *S. aureus*, while *P. aeruginosa* is most frequently isolated from adult CF patients (Cystic Fibrosis Foundation, 2014). It has been established that different species of microbes interact in the airways of CF patients, resulting in a pathogenesis different from a single-species infection. *S. aureus* and *P.aeruginosa* is the predominant combination found in CF patients. Interspecies as well as intraspecies interaction has been observed in CF patients.

#### 4.4.1 Virus and bacteria cooperativity in CF

Rhinovirus, RSV, Influenza A and B are the most common viruses detected in CF patients, while *S. aureus* and *P. aeruginosa* are the most common bacterial pathogens. RSV-induced elevated bacterial adherence in CF is achieved through the direct binding of RSV to *P. aeruginosa* via RSV glycoprotein G (Van Ewijk et al., 2007). Other mechanisms of interaction between RSV and *P.aeruginosa* include viral-induced increased biofilm growth (Hendricks et al., 2016; Kiedrowski et al., 2018), viral-induced biofilm dispersion by production of H_2_O_2_ (Chattoraj et al., 2011a), altered immune response, etc.

Conversely, prior *P. aeruginosa* infection has been found to modulate antiviral response significantly. Chattoraj et al. (2011b) found higher viral load in co-infected cells compared to RV infection alone. Sörensen et al. (2020) found that *P. aeruginosa* blocks the antiviral IFN signaling via LasR-dependent degradation of IFNλ by protease AprA. Endres et al. (2022) also found that non-mucoid *P. aeruginosa* significantly controls the innate antiviral response, creating a favorable environment for RV infection. For example, IL-6 was almost completely degraded by bacterial protease, and the epithelial barrier function was also compromised. Bomberger et al. (2014) found that the *P. aeruginosa* protein, Cif, enhanced viral infection of CF epithelial cells by preventing MHC class I antigen presentation and CD8 T cell-mediated removal of influenza A-infected cells.

### 4.5 SARS-CoV-2 infections (COVID-19)

Various studies suggested that the percentage of COVID-19 patients with co-infections varies greatly, from 0 to 100 percent in those who die, and antimicrobial use varies widely by the severity of the disease, ranging from 20 to 100 percent for antibiotics (Langford et al., 2020). Before COVID-19, Zahariadis et al. (2006) had identified co-infections of pulmonary microbes in patients diagnosed with SARS; 9% were caused by *M. pneumonia*, while 30% were by *C. pneumonia*. In a study carried out at a hospital in Wuhan, China, Zhang et al. (2020) found that out of all the 221 patients with COVID-19 pneumonia that were admitted, co-infection occurred in 57 of them and again among these 57 patients, 17 of them were confirmed to be caused by bacterial-co-infection. A systemic review of thirty studies involving more than 3,000 patients found that 7% have bacterial co-infection (Lansbury et al., 2020). Another systemic review by Langford et al. (2020) identified secondary bacterial co-infection in 14.3% of the patients. The important bacteria detected in COVID-19 co-infection include *S. pneumoniae*, *S. aureus, Legionella pneumophila*, *M. pneumonia*, *A. baumanii, Enterobacter cloaca, K. pneumonia* (Chen et al., 2020; Duployez et al., 2020; Wang et al., 2020; Yu et al., 2020). Secondary bacterial infection in COVID-19 patients often exacerbates the disease, resulting in more extended hospitalization and even mortality in many cases. Zhou et al. (2020) suggested that 50% of COVID-19 deaths were due to bacterial co-infections.

#### 4.5.1 Virus and bacteria cooperativity in COVID-19

The interaction mechanism between COVID-19 and respiratory tract bacteria is similar to bacterial interactions with viruses like RSV, Influenza, etc. One mechanism by which virus-bacteria co-infection occurs is due to the host’s immune system dysfunction, which can be weakened due to viral infection, thus promoting viral-bacterial co-infection. COVID-19, in this case, is a good example in which bacterial co-infection is identified in most hospitalized COVID-19 patients. The COVID-19 patients were found to acquire increased levels of inflammatory cytokines and biomarkers, indicating that the dysregulation of the immune system paved the way for secondary bacterial infection (Tetro, 2020). Another common interaction mechanism between bacteria and viruses is the enhancement of bacterial adhesion to epithelial cells via the upregulation of surface receptors. Golda et al. (2011) found that prior human coronavirus NL63 infection increases the expression of surface receptors like PAFR in the human epithelial cells. This enhances the adherence of *S. pneumoniae* to human epithelial cells. It has been shown that SARS-CoV-2 infection damages the lung epithelial cells and the lung infrastructure, recruiting a number of immune cells, such as macrophages and neutrophils, which cause inflammation and eventually facilitate bacterial invasion and adherence (MacIntyre et al., 2018; Vellingiri et al., 2020). Feldman and Anderson (2021) suggested that severe immunosuppression by SARS-CoV-2 infection may also activate the quiescent airway pathogens like *S. pneumoniae, S. aureus*, and *H. influenzae* enclosed in biofilms. Increased cytokine production by SARS-CoV-2 may reactivate latent or facilitate active TB development (Al Lawati et al., 2021). The prevalence of co-infection can also be attributed to the fact that hospital-associated bacteria can quickly adapt to the host environment of immunocompromised patients (Mirzaei et al., 2020). It has also been hypothesized that the accumulation of H_2_O_2_ in the lungs due to SARS-CoV-2 infection may suppress the innate immune response, promoting secondary bacterial infection (Bayindir and Bayindir, 2020). However, experimental studies have yet to be performed to prove this hypothesis.

## 5 Conclusion

The human respiratory tract inhabits a complex microbial community composed of commensal and opportunistic pathogens, the most common being bacteria such as *S. aureus*, *S. pneumoniae*, and *M. catarrhalis*, along with Influenza virus, Rhinovirus, SARS-CoV-2, etc. There is a synergistic and competitive interaction among these pathogens. Typically, respiratory tract microbiota causes no harm to the body, and homeostasis exists between formal micro-flora and commensal and opportunistic pathogens. However, alterations or dysbiosis in this community may result in the attainment of bacterial or viral pathogens and displacement of opportunistic pathogens to harmful pathogens, resulting in pathogenic invasion. Respiratory viruses such as influenza, Rhinovirus, and SARS-CoV-2 could cause bacterial superinfections by triggering opportunistic bacteria in the respiratory tract. As a result of bacterial co-infections, there is a rise in the morbidity rate, and subsequently, the mortality rate has significantly increased in patients with viral infections. This interplay between virus and bacteria is complex, and in order to predispose co-infections, many components, including the bacteria, virus, and the host’s immune system, are involved. The mechanism by which viruses can trigger bacterial co-infection is by damaging the respiratory epithelial cells, exposing the bacterial receptors, damaging the cilia, altering the mucosal environment, dysregulation of the immune system, changing the function of phagocytes, promoting the release of inflammatory mediators such as chemokines, cytokines release, overexpression of antimicrobial peptides and by enhancing bacterial adherence. With the onset of the current COVID-19 pandemic comes a new surge of interest in understanding the relationship between respiratory bacteria and the virus. From several experiments and observations, it is clear that the interaction between viruses and bacteria could aggravate infections and even enhance secondary infections before existing diagnosis. Since the co-infection included bacteria and viruses, an effective strategy is required to manage the infection.

## Author contributions

CL: Conceptualization, Investigation, Writing – original draft, Writing – review and editing, Methodology. MY: Conceptualization, Supervision, Writing – review and editing, Investigation, Writing – original draft. PS: Conceptualization, Supervision, Writing – review and editing. AS: Conceptualization, Writing – review and editing. MI: Writing – review and editing, Supervision. BV: Conceptualization, Methodology, Supervision, Writing – review and editing. RZ: Writing – review and editing. ZP: Writing – review and editing, Supervision. HR: Writing – review and editing, Supervision, Methods.
